# The immunoglobulin‐like domain of neuregulins potentiates ErbB3/HER3 activation and cellular proliferation

**DOI:** 10.1002/1878-0261.12310

**Published:** 2018-05-14

**Authors:** Ariana Centa, Ruth Rodríguez‐Barrueco, Juan Carlos Montero, Atanasio Pandiella

**Affiliations:** ^1^ Instituto de Biología Molecular y Celular del Cáncer IBSAL CSIC and CIBERONC Salamanca Spain; ^2^ Present address: Departament de Patologia i Terapèutica Experimental Facultat de Medicina i Ciències de la Salut IDIBELL Hospitalet de Llobregat Universitat de Barcelona Barcelona Spain

**Keywords:** breast cancer, HER receptors, Ig‐like domain, neuregulins

## Abstract

The neuregulins (NRGs) represent a large family of membrane‐anchored growth factors, whose deregulation may contribute to the pathogenesis of several tumors. In fact, targeting of NRG‐activated pathways has demonstrated clinical benefit. To improve the efficacy of anti‐NRG therapies, it is essential to gain insights into the regions of NRGs that favor their pro‐oncogenic properties. Here, we have addressed the protumorigenic impact of different NRG domains. To do this, deletion mutants affecting different NRG domains were expressed in 293 and MCF7 cells. Of the five forms studied, only the wild‐type and a mutant lacking the Ig‐like domain (NRG^ΔIg^) were properly sorted to the plasma membrane. Both forms were released as soluble forms to the culture media. However, the mutant NRG^ΔIg^ failed to efficiently activate HER2 and HER3 receptors, signaling pathways, and cell proliferation when compared to wild‐type NRG. Treatment with trastuzumab, a humanized antibody used in the breast cancer clinic, inhibited the constitutive activation of HER2, HER3, and downstream signaling in MCF7 cells constitutively expressing wild‐type NRG. In contrast, this treatment had a marginal effect on MCF7‐NRG^ΔIg^ cells. This study demonstrates that the Ig‐like region of NRGs exerts an important role in their capability to activate ErbB/HER receptors and mitogenic responses. Strategies aimed at targeting NRGs should consider that fact to improve neutralization of the pro‐oncogenic properties of NRGs.

**Abbreviations**
ADAM: a disintegrin and metalloprotease
AKT: protein kinase B (PKB)
EGF: epidermal growth factor
ERK1/2: extracellular signal‐regulated protein kinases 1 and 2
GAPDH: glyceraldehyde‐3‐phosphate dehydrogenase
GSK3‐α/β: glycogen synthase kinase‐3 alpha and beta
HER/ErbB: human epidermal growth factor receptor
MEK1/2: dual specificity mitogen‐activated protein kinase kinase 1 and 2 (MAP2K1)
NDRG1: N‐myc downstream regulated 1
NRG: neuregulin
PKC: protein kinase C
PMA: phorbol 12‐myristate 13‐acetate
S6: ribosomal protein S6
TACE: tumor necrosis factor‐α‐converting enzyme
TGFα: transforming growth factor‐alpha

## 1 Introduction

The neuregulins (NRGs) are a group of polypeptide growth factors of the epidermal growth factor (EGF) family which participate in various physiological processes such as heart and peripheral nervous system development (Breuleux, 2007; Britsch *et al*., 1998; Falls, 2003; Massague and Pandiella, 1993; Meyer and Birchmeier, 1995; Montero *et al*., 2008). The NRGs act by binding to the extracellular region of the ErbB/HER transmembrane tyrosine kinases, particularly HER3 and HER4 (Burden and Yarden, 1997; Carraway and Burden, 1995). Upon binding of NRGs to their extracellular region, the receptors dimerize, especially with HER2 (Burden and Yarden, 1997; Carraway and Burden, 1995; Jeong *et al*., 2014), and this leads to activation of a complex network of downstream signaling events (Esparis‐Ogando *et al*., 2016).

Four different NRG genes code for more than 30 different isoforms, most of them synthesized as membrane‐bound forms, generically termed proNRGs (Falls, 2003; Hayes *et al*., 2007; Montero *et al*., 2008). Because of their transmembrane disposition, the proNRGs are constituted by three blocks: the N‐terminal extracellular domain, the transmembrane domain, and the C‐terminal intracellular region. The N‐terminal extracellular block includes the EGF‐like module, together with other regions such as Ig‐like or the kringle‐like subdomains (Falls, 2003; Wen *et al*., 1994). The EGF‐like domain is critical for HER receptor binding and activation (Holmes *et al*., 1992; Wen *et al*., 1994), while the Ig‐like domain appears to favor interaction with extracellular matrix components (Loeb and Fischbach, 1995; Loeb *et al*., 1999). The role of the Ig‐like domain in regulating the interaction of NRGs with their receptors is controversial. In fact, while some studies have indicated that the Ig‐like region may promote the interaction of soluble NRGs with their receptors, favoring activation of the latter (Eto *et al*., 2006), other studies suggested that such domain conferred signal attenuation by inducing downregulation of ErbB/HER receptors (Warren *et al*., 2006). The ectodomain of the proNRGs is connected to an internal hydrophobic domain that acts as both a transmembrane domain and a signal sequence (Falls, 2003). That transmembrane region is followed by an intracellular domain which varies in length among the different NRG isoforms, but appears to be required for the adequate sorting of proNRGs to the plasma membrane (Montero *et al*., 2011).

The membrane‐bound proNRG forms may undergo proteolytic processing to generate soluble forms of the factor (Falls, 2003; Montero *et al*., 2000). Such process is slow under resting conditions, but may accelerate under situations in which intracellular signaling pathways are stimulated (Montero *et al*., 2000, 2002). In fact, activation of routes that increase phosphorylation of cellular proteins provokes ectodomain cleavage of proNRGs, generating soluble NRGs (Montero *et al*., 2000, 2002). Such proteolytic event is accomplished by membrane metalloproteases of the ADAM subfamily, especially by ADAM17 (also termed TACE) or ADAM10 (Ebbing *et al*., 2016; Montero *et al*., 2000).

Deregulation of NRGs has been linked to important pathological processes such as cancer or schizophrenia (Breuleux, 2007; Mei and Xiong, 2008; Montero *et al*., 2008). In fact, several studies suggested that NRGs play a role in the genesis or progression of breast tumors. Thus, expression of NRGs in the mammary gland of mice provoked the appearance of breast adenocarcinomas (Krane and Leder, 1996). Moreover, increased expression of NRGs has been described in up to 50% of human breast tumors, and such increased expression has been linked to poor patient outcome (de Alava *et al*., 2007). That negative impact on the clinical evolution of patients bearing NRG+ tumors may be related to the pro‐oncogenic properties of NRGs, which include promotion of cell proliferation, migration, or metastatic dissemination (Atlas *et al*., 2003; Seoane *et al*., 2016; Tsai *et al*., 2003; Yuste *et al*., 2005). Moreover, some reports have linked NRG expression to the effectiveness of certain antitumoral therapeutics (de Alava *et al*., 2007). Thus, NRG expression in breast tumors has been shown to biomark sensitivity to the anti‐HER2 therapeutic antibody trastuzumab (Meetze *et al*., 2015). In addition, NRGs may provoke resistance to certain therapies used to target HER2 (Schwarz *et al*., 2017; Yang *et al*., 2017).

Those evidences raise the possibility that NRG targeting may be therapeutically useful in situations in which NRGs play a pro‐oncogenic role favoring tumor growth or dissemination. In fact, and in addition to the above‐mentioned evidences obtained in breast cancer, a recent report has demonstrated the clinical value of targeting NRG–HER system in patients with tumors containing *NRG1* gene rearrangements (Jones *et al*., 2017; Kim *et al*., 2016). Interestingly, some of these rearrangements excluded parts of the N‐terminal region of *NRG1*‐derived gene products whose biological function(s) are still poorly known (Eto *et al*., 2006; Kim *et al*., 2016). Reasonably, a better knowledge of the biological properties of the different proNRG domains may help in designing adequate anti‐NRG strategies. Considering this, and to gain additional insights into the relevance of different proNRG domains in their biological action, we prepared several deletion mutants and explored their role in regulating proNRG signaling. We show that elimination of the Ig‐like domain of proNRGs strongly affected the HER receptor activating properties of soluble and membrane‐anchored proNRGs, which translated into poor biological activity of NRG forms that lack the Ig‐like region.

## 2 Materials and methods

### 2.1 Reagents and immunochemicals

Culture media, fetal bovine serum, trypsin, penicillin, and streptomycin were from GIBCO BRL (Gaithersburg, MD, USA). Protein A‐Sepharose, proteinase K, phorbol 12‐myristate 13‐acetate (PMA), doxycycline, 4′,6‐diamidino‐2‐phenylindole (DAPI), and 3‐(4,5‐dimethylthiazol‐2‐yl)‐2,5‐diphenyltetrazolium bromide (MTT) were from Sigma‐Aldrich (St Louis, MO, USA). Immobilon^®^‐P (PVDF) transfer membrane, Immobilon^®^‐FL membranes, and Amicon^®^ Ultra Centrifugal Filters were from Merck Millipore Corp. (Darmstadt, Germany). Human recombinant neuregulin‐1 was from ProSpec Protein Specialists (Rehovot, Israel). Other generic chemicals were purchased from Sigma‐Aldrich, Roche Biochemicals (Barcelona, Spain), or Merck (Darmstadt, Germany).

The rabbit anti‐NRG endodomain and ectodomain antibodies as well as the anti‐HER3 and anti‐HER4 antibodies have been formerly described (Montero *et al*., 2000, 2007; Sanchez‐Martin and Pandiella, 2012). Trastuzumab and pertuzumab were purchased from a local pharmacy. The rabbit polyclonal antibodies to phospho‐HER2 (Tyr1221/1222), AKT, phospho‐MEK1/2 (Ser217/221), MEK1/2, phospho‐S6 ribosomal protein (Ser240/244), phospho‐NDRG1 (Thr346), and phospho‐GSK3‐α/β (Ser 21/9) and the rabbit monoclonal antibodies to phospho‐HER3 (Tyr1289) and S6 ribosomal protein were obtained from Cell Signaling Technologies (Beverly, MA, USA). The mouse monoclonal anti‐phospho‐ERK1/2 (Tyr204), anti‐glyceraldehyde‐3‐phosphate dehydrogenase (GAPDH), anti‐phospho‐tyrosine (PY99) and the rabbit polyclonal anti‐ERK1/2 antibodies were from Santa Cruz Biotechnology (Santa Cruz, CA, USA). The mouse monoclonal anti‐HER2 (clone Ab‐3) was from Calbiochem‐Behring Corp. (San Diego, CA, USA). The mouse monoclonal anti‐phospho‐AKT (Ser473) was from BD Pharmingen (Palo Alto, CA, USA). The rabbit polyclonal anti‐calnexin was from Stressgen Bioreagents (Victoria, BC, Canada). The secondary horseradish peroxidase (HRP)‐conjugated antibodies, anti‐rabbit, anti‐rabbit light chain, anti‐rabbit conformation‐specific, and anti‐mouse, were from Bio‐Rad Laboratories (Hercules, CA, USA), Jackson ImmunoResearch Laboratories (West Grove, PA, USA), Cell Signaling Technologies, and GE Healthcare Life Sciences (Piscataway, NJ, USA). The Cy3‐conjugated secondary antibody was from Jackson ImmunoResearch (West Grove, PA, USA). The anti‐mouse DyLight™ 680‐conjugated and anti‐rabbit DyLight™ 800‐conjugated antibodies were from Thermo Fisher Scientific (Waltham, MA, USA).

### 2.2 Cell culture and transfections

MCF7 and 293 cells were grown in Dulbecco's modified Eagle's medium (DMEM) supplemented with 10% fetal bovine serum (FBS), containing high glucose (4500 mg·L^−1^) and antibiotics (penicillin 100 U·mL^−1^, streptomycin 100 mg·mL^−1^). Cell lines were cultured at 37°C in a humidified atmosphere in the presence of 5% CO_2_ and 95% air.

The plasmids coding for rat proNRGα2c and the proNRG^ΔIg^, proNRG^Δintra^, proNRG^Δextra^, and NRG^β3^ mutants were transfected into 293 or MCF7 cells by calcium phosphate or using Lipofectamine (Invitrogen, Life Technologies, Carlsbad, CA, USA), respectively. Clones expressing these constructions were selected by G418 resistance and their NRG expression analyzed by western blotting (Montero *et al*., 2000, 2007, 2011).

### 2.3 Protein extraction, immunoprecipitation, and western blotting

Detailed procedures for the extraction, quantitation, and immunoprecipitation of proteins and western blotting can be consulted in Montero *et al*. (2011).

### 2.4 Production of soluble NRG

293 and MCF7 cells expressing the wild‐type proNRGα2c and the different mutants were plated in 100‐mm dishes and cultured in DMEM with 10% FBS up to 80% confluency. The medium of the cells was replaced with DMEM without FBS and incubated for 30 min. This process was repeated three times. Then, cells were incubated with 5 mL of DMEM without FBS for 24 h. Conditioned medium was 10× concentrated by ultrafiltration using Amicon^®^ 3K Ultra Centrifugal Filters (Merck Millipore Corp.).

### 2.5 Proteinase protection experiments

293 and MCF7 cells expressing the proNRGα2c or proNRG^ΔIg^ were washed once with Krebs/Ringer/Hepes buffer (containing, in mmol·L^−1^: NaCl, 140; KCl, 5; CaCl_2_, 2; MgSO_4_, 1.2; KH_2_PO_4_, 1.2; glucose, 6; and Hepes, 25, pH 7.4) and then incubated in this buffer supplemented with 200 μg·mL^−1^ proteinase K for 30 min at room temperature. Cells were then washed three times with PBS containing 2 mm PMSF and lysed in 1 mL of lysis buffer with protease and phosphatase inhibitors.

### 2.6 Bioactivity assays

The conditioned media of 293 cells expressing proNRGs were used to stimulate monolayers of MCF7 cells for 15 min. On the other hand, clones of MCF7^tetoff^ expressing proNRGα2c and proNRG^ΔIg^ were cultured in DMEM with 10% FBS for up to 40% confluency, after the cells were serum‐starved and treated with or without doxycycline (10 ng·mL^−1^) for 48 h. MCF7^tetoff^ cells expressing proNRGα2c and proNRG^ΔIg^ were plated in 24‐well plates to a density of 15 000 cells/well and cultured overnight in DMEM with 10% FBS. The next day, cells were treated with or without 10 ng·mL^−1^ doxycycline for 48 h. Later, cells were serum‐starved and maintained with or without doxycycline (10 ng·mL^−1^) and treated with or without NRG‐1 human recombinant (10 nm). After 5 days, the MTT uptake assay was performed (Esparis‐Ogando *et al*., 2002). MCF7 cells were plated in 24‐well plates to a density of 15 000 cells/well and cultured overnight in DMEM + 10% FBS. The next day, the medium was replaced with DMEM without FBS containing conditioned media of 293, 293‐NRGα2c, and 293‐NRG^ΔIg^ cells. Cell proliferation was analyzed at 5 days by an MTT‐based assay (Esparis‐Ogando *et al*., 2002). On the other hand, clones of MCF7^tetoff^ expressing proNRGα2c and proNRG^ΔIg^ were plated in 6‐well plates to a density of 30 000 cells/well and were cultured in DMEM with 10% FBS with or without doxycycline (10 ng·mL^−1^) for 48 h. Later, the medium was replaced by DMEM with 1% FBS and treated with or without doxycycline (10 ng·mL^−1^) and trastuzumab (10 nm) for 5 days. At the end of incubation, the cells were counted in Z1™ Coulter Particle Counter^®^ (Beckman Coulter™ Life Sciences, Indianapolis, IN, USA).

### 2.7 Immunofluorescence

The immunofluorescence protocol has been described (Esparis‐Ogando *et al*., 2002). Dilutions of the anti‐NRG endodomain or ectodomain antibodies were 1 : 500.

### 2.8 Quantitative and statistical analyses

Quantitation of the bands obtained from western blotting experiments was performed using the imagej 1.44 software (National Institutes of Health, Bethesda, MD, USA) or the odyssey infrared imaging system V.3.0 (LI‐COR, Lincoln, NE, USA). The intensity of each of the different bands was calculated with respect to the control, and the data are represented as the percentage of the maximum value obtained for each experiment.

Results obtained in the proliferation experiments are represented as the mean ± standard deviation (SD) of triplicates of a representative experiment that was repeated at least three times using ibm spss statistics 24 (Madrid, Spain), and differences were tested for significance using Student's *t*‐test. Significance was considered when *P* values were < 0.05.

## 3 Results

### 3.1 Impact of different proNRG domains on the production of soluble NRG

**Figure 1:**
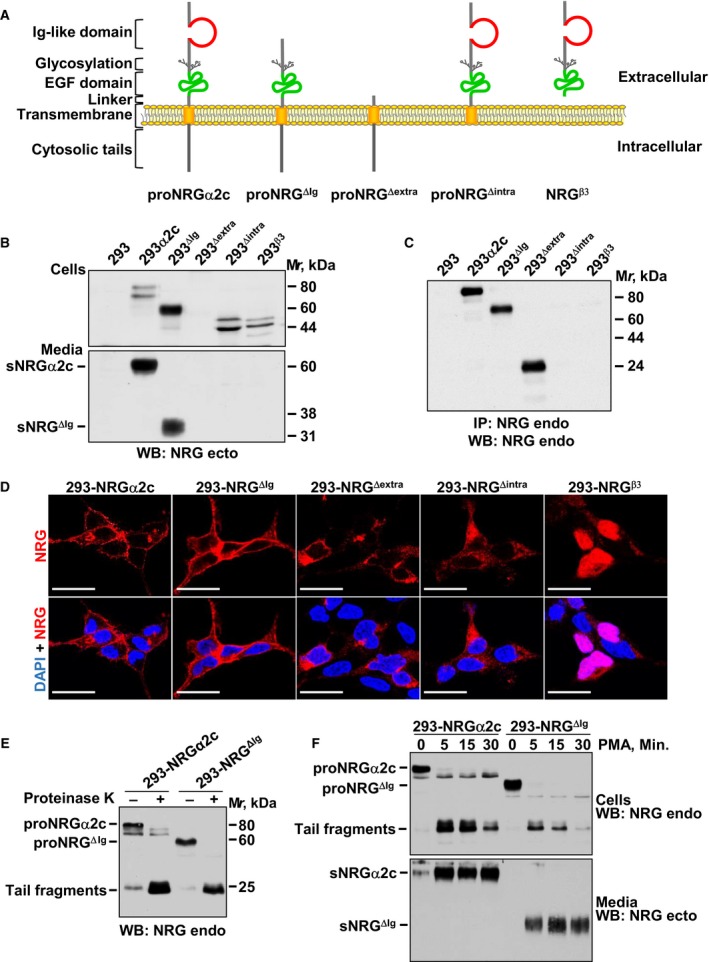
Subcellular localization of NRGα2c and different mutants. (A) Schematic representation of domains of proNRGα2c and distinct deletion mutants of the isoform wild‐type. (B, C) Expression of proNRGα2c and different mutants in 293 cells. 293 cells expressing these proteins were lysed, and the expression of NRG analyzed by western blotting using the antibody that recognizes the intracellular domain (anti‐NRG endo) (C) or extracellular domain (anti‐NRG ecto) (B, top panel). The conditioned medium of these cells was collected, and the expression of sNRG was analyzed by western blotting using the anti‐NRG ecto (B, bottom panel). (D) Immunofluorescence analysis of the subcellular distribution of proNRGα2c and the different mutants in 293 cells. The localization of proNRGα2c, proNRG^ΔIg^, and proNRG^Δextra^ was assessed with the anti‐NRG endo antibody and the distribution of proNRG^Δintra^ and NRG^β3^ was analyzed with the anti‐NRG ecto antibody as described in Materials and methods. Images were captured using a Leica TCS SP5 confocal microscope (Barcelona, Spain). Bar, 25 μm. (E) Protease protection experiments of the 293‐NRGα2c and 293‐NRG^ΔIg^ cells. These cells were treated with or without proteinase K (200 μg·mL^−1^) for 30 min, and the expression of NRG was analyzed by western blotting using anti‐NRG endo antibody. (F) Effect of PMA in the cleavage of proNRG. 293‐NRGα2c and 293‐NRG^ΔIg^ cells were treated with PMA at the indicated times. The expression of NRG in cells extract (top panel) or conditioned medium (bottom panel) was analyzed by western blotting with the specified antibodies.

To explore the relevance of different domains of proNRGs in their biological action, distinct deletion mutants of the isoform proNRGα2c, derived from the *NRG1* gene, were prepared (Fig. 1A). Such isoform was chosen as it has been extensively used to analyze biological characteristics of proNRGs (Montero *et al*., 2007, 2011). The proNRGα2c mutants constructed included deletions in (a) the Ig‐like domain (proNRG^ΔIg^), (b) the ectodomain (proNRG^Δextra^), (c) the endodomain (proNRG^Δintra^), and (d) the transmembrane and intracellular domains, generating a form that mimics NRG^β3^. Wild‐type proNRGα2c and the different mutants were transfected into 293 cells, and their expression was evaluated by western blotting. In cell lysates, and using an antibody raised to the NRG/EGF‐like region of the ectodomain, that antibody recognized wild‐type proNRGα2c, proNRG^ΔIg^, proNRG^Δintra^, and NRG^β3^ (Fig. 1B, top panel). The antibody failed to recognize the proNRG^Δextra^ form that lacks the extracellular region. Expression of proNRG^Δextra^ form was detected using an antibody that recognizes the intracellular region of proNRGα2c (Fig. 1C).

To assess the production of soluble forms of NRGs (sNRG), culture media were harvested and concentrated and NRG analyzed by western blotting using the anti‐NRG ectodomain antibody. These experiments demonstrated that proNRGα2c and proNRG^ΔIg^ were able to release soluble forms to the culture media (Fig. 1B, bottom panel). The molecular weights of the two soluble forms differed because of the deletion of the Ig‐like region in sNRG^ΔIg^. Soluble NRG forms derived from proNRG^Δintra^ or from NRG^β3^ were undetectable in the culture media. As expected, soluble NRG was not detected in the proNRG^Δextra^ as this mutant lacks the NRG/EGF‐like domain that contains the epitope recognized by the antibody.

### 3.2 proNRGα2c and proNRG^ΔIg^ are properly sorted to the plasma membrane and processed

The different capability of 293 cells expressing the distinct NRG forms to generate soluble forms of NRGs led us to explore the reason for such differences. Immunofluorescence studies indicated that the failure of cells expressing proNRG^Δintra^ or NRG^β3^ to release sNRG to their culture media was likely due to their entrapment in intracellular compartments (Fig. 1D). ProNRG^Δintra^ accumulated in a perinuclear intracellular region, while NRG^β3^ colocalized with the nuclear stain DAPI, in agreement with previously reported results. ProNRG^Δextra^ also accumulated intracellularly, especially in a perinuclear region. The lack of cell surface staining in the case of proNRG^Δextra^ and proNRG^Δintra^ confirms that these domains are required for transport of proNRGs to the plasma membrane (Montero *et al*., 2011).

These immunofluorescence studies showed cell surface staining of proNRGα2c and proNRG^ΔIg^, suggesting that proNRG^ΔIg^ reached the cell surface as well as wild‐type proNRGα2c. To verify that proNRG^ΔIg^ reached the plasma membrane correctly, protease protection experiments were carried out. 293‐NRGα2c and 293‐NRG^ΔIg^ cells were treated with proteinase K, and then, cell extracts were analyzed by western blotting using the anti‐endodomain antibody. As shown in Fig. 1E, treatment of intact cells with proteinase K caused a profound decrease in transmembrane, cell‐bound proNRGα2c. That effect was accompanied by the concomitant generation of fragments with molecular weights of ≈ 25 kDa. The latter represent cell‐bound truncated fragments of proNRGs which include the transmembrane and cytosolic domains. In the case of 293‐NRG^ΔIg^ cells, the slow‐migrating mature form was sensitive to treatment with proteinase K, and treatment with the protease resulted in generation of the 25‐kDa tail fragments. The above results indicate that the presence of the immunoglobulin domain is not essential for proNRGs to be transported to the cell membrane.

We also analyzed the relevance of the Ig‐like domain on the cleavage of transmembrane proNRG^ΔIg^. Regulated cleavage of membrane‐anchored growth factors may occur by activation of several signaling pathways, including the protein kinase C (PKC) route (Montero *et al*., 2002). Treatment with the PKC activator PMA caused a decrease in cell‐associated NRG and a concomitant increase in membrane‐associated tail fragments in both 293‐NRGα2c cells and 293‐NRG^ΔIg^ cells (Fig. 1F). Parallel analyses of culture media showed accumulation of soluble NRGα2c and NRG^ΔIg^ in cells treated with PMA.

### 3.3 The Ig‐like domain of NRGs facilitates activation of HER receptors

**Figure 2:**
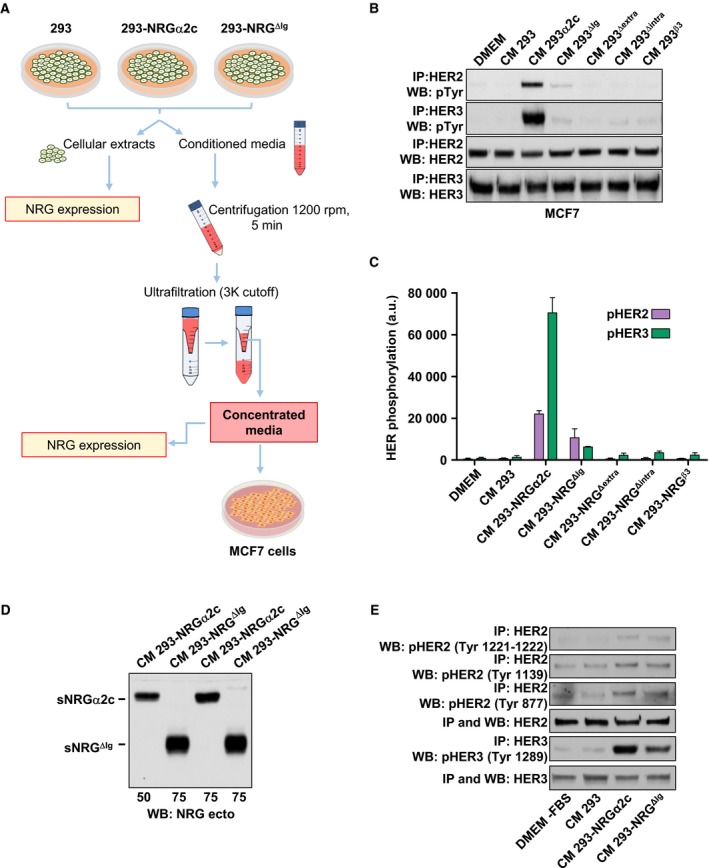
Effect of soluble NRG on activation of HER receptors. (A) Schematic representation of bioactivity assay of the soluble NRG. The conditioned medium of the 293 cells expressing NRGα2c and NRG^ΔIg^ was collected and concentrated. The levels of NRG in cell extracts or released into the medium were detected by western blotting. This conditioned medium was used for stimulated the MCF7 cells. (B) Effect of conditioned medium on activation of HER receptors. MCF7 cells stimulated with the conditioned medium of 293 cells expressing the NRGα2c and different mutants for 15 min were lysed. One milligram of extract was used to immunoprecipitated HER2 and HER3 and their tyrosine phosphorylation and total levels were detected by western blotting. The conditioned medium of 293 cells was used as a negative control. (C) Bar graph representing the quantification of HER2 and HER3 phosphorylation in MCF7 cells stimulated with the conditioned medium of the 293 cells expressing different mutants of NRG. Data are presented as the mean ± SD of three independent experiments performed as in (B). (D) The levels of sNRG released into the medium of 293‐NRGα2c and 293‐NRG^ΔIg^ cells were analyzed by western blotting using anti‐NRG ectodomain. The volume of conditioned medium load in the gel is equivalent to the amount in micrograms of cellular extracts (indicated in the bottom of panel). (E) HER2 and HER3 phosphorylation in MCF7 cells stimulated with the conditioned medium of 293‐NRGα2c and 293‐NRG^ΔIg^ cells**.** The phosphorylation of HER2 and HER3 in tyrosine‐specific residues was evaluated by western blotting using phosphospecific antibodies.

The HER‐activating capability of the culture media from cells expressing the different NRG forms was then evaluated. For these experiments, 293 cells were cultured for 24 h in their growth media, which was then harvested and after concentration was added to monolayers of MCF7 cells (Fig. 2A). MCF7 cells express the NRG receptors HER3 and HER4 which may oligomerize with other HER receptors, especially HER2, facilitating transphosphorylation of HER3, HER4, and HER2. This binary cellular system has been used to explore paracrine signaling by soluble NRGs. Media derived from 293 cells expressing wild‐type proNRGα2c caused phosphorylation of HER2 and HER3 (Fig. 2B). Quantitative analyses indicated higher tyrosine phosphorylation of HER3 receptors as compared to HER2 receptors (Fig. 2C). Phosphorylation of HER4 was difficult to detect due to the low level of expression of this receptor in MCF7 cells (data not shown). Media harvested from cells expressing the proNRG^ΔIg^ were much less efficient in their capability to induce HER2 and HER3 phosphorylation (Fig. 2B,C). Media recovered from proNRG^Δintra^ or from NRG^β3^ cells failed to activate HER2 or HER3 (Fig. 2B,C), in line with the failure to detect sNRG in the culture media harvested from 293 cells transfected with these mutants (Fig. 1B). Titration experiments demonstrated that the lower HER receptor tyrosine phosphorylation efficiency of conditioned media from cells expressing proNRG^ΔIg^ was not due to lower amounts of sNRG^ΔIg^ being collected from the culture media (Fig. 2D).

As these western blotting experiments were performed with a generic anti‐pY antibody, the possibility that the pY signal detected in the western blots was not exclusively due to HER2 or HER3 in their respective immunoprecipitates was contemplated. In fact, coprecipitation of HER2 and HER3 has been reported in MCF7 cells, particularly when treated with NRG (Sanchez‐Martin and Pandiella, 2012). To explore the effect of the conditioned media from 293 cells expressing proNRGα2c and proNRG^ΔIg^ on pHER2 and pHER3, lysates from MCF7 cells incubated with conditioned media harvested from both cell lines were analyzed by western blotting using antibodies specific for phosphotyrosine residues present in HER2 or HER3. As shown in Fig. 2E, immunoprecipitation with anti‐HER2, followed by western blotting with antibodies that recognize pY1221–1222, pY1139, or pY877 in HER2, gave weak tyrosine phosphorylation signals. A much higher signal was observed when HER3 was immunoprecipitated and western blots were probed with an anti‐pHER3 pY1289 antibody. Together, these results indicate that soluble NRGs derived from the conditioned media of cells expressing proNRGα2c or proNRG^ΔIg^ principally upregulate tyrosine phosphorylation of HER3 in MCF7 cells.

### 3.4 Signaling responses to soluble NRGα2c and NRG^ΔIg^

**Figure 3:**
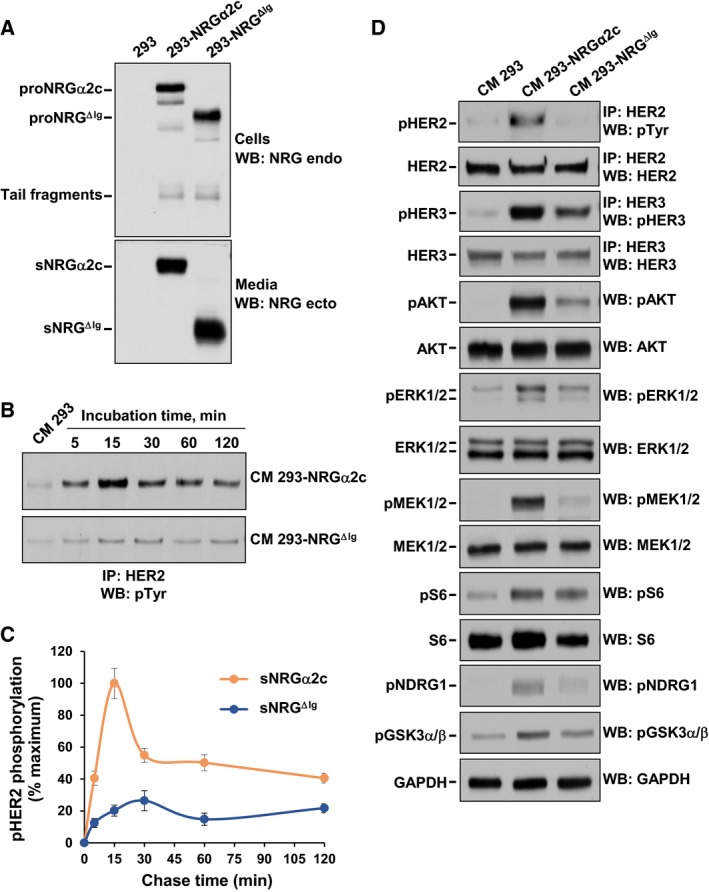
Relevance of the Ig‐like domain in the signaling of HER receptors. (A) Expression of the NRG in cell extract (top panel) and conditioned medium (bottom panel) of the 293, 293‐NRGα2c, and 293‐NRG^ΔIg^ cells analyzed by western blotting. (B) Time course of the effect of conditioned medium of 293‐NRGα2c and 293‐NRG^ΔIg^ cells on HER2 phosphorylation in MCF7 cells. The conditioned medium of 293 cells was used as a negative control. (C) Quantification of HER2 phosphorylation in MCF7 cells stimulated with conditioned medium of 293‐NRGα2c and 293‐NRG^ΔIg^ cells. Data were relativized to maximal phosphorylation obtained and are represented as the mean ± SD of two independent experiments performed as in (B). (D) Effect of conditioned medium of 293‐NRGα2c and 293‐NRG^ΔIg^ cells in the signaling of HER receptors. HER2 and HER3 phosphorylation and the activation of proteins in HER signaling pathway were evaluated by western blotting with the antibodies indicated in the left side of figure.

Considering the differences found in the tyrosine phosphorylation of HER2 and HER3 by sNRGα2c or sNRG^ΔIg^, how the presence of the Ig‐like domain could influence signaling by these receptors was analyzed. For these experiments, clones of cells expressing similar protein levels in their transmembrane form and which released similar amounts of sNRGα2c or sNRG^ΔIg^ were selected (Fig. 3A). Time‐course experiments were performed to obtain information about the incubation times to reach maximum HER receptor activation. These studies showed that tyrosine phosphorylation of HER2 receptors caused by conditioned media containing sNRGα2c or sNRG^ΔIg^ followed analogous time courses (Fig. 3B,C), even though HER2 phosphorylation in response to addition of sNRG^ΔIg^ conditioned media was lower than that of wild‐type NRGα2c (Fig. 3B,C). Maximum HER2 phosphorylation was reached within 15 min of incubation with the conditioned media.

To explore whether the differential activation of HER receptors by sNRGα2c and sNRG^ΔIg^ translated into differences in signaling, MCF7 cells were incubated for 15 min with the concentrated medium collected from 293 cells transfected with proNRGα2c and proNRG^ΔIg^. The levels of expression and phosphorylation of HER2 and HER3 receptors and proteins that serve as readouts of pathway activation were analyzed (Fig. 3D). These studies confirmed that the phosphorylation of HER2 and HER3 by conditioned media from 293 cells expressing proNRGα2c was much more potent than their activation by conditioned media from cells expressing proNRG^ΔIg^. Analyses of proteins that act in HER signaling demonstrated that the presence of the Ig‐like domain favored the signaling activity of sNRG. All evaluated proteins were activated by both forms of sNRG, but had a higher phosphorylation after incubation with the medium from 293‐NRGα2c cells as compared to the medium from 293‐NRG^ΔIg^ cells.

### 3.5 Biological activity of proNRGα2c and proNRG^ΔIg^

**Figure 4:**
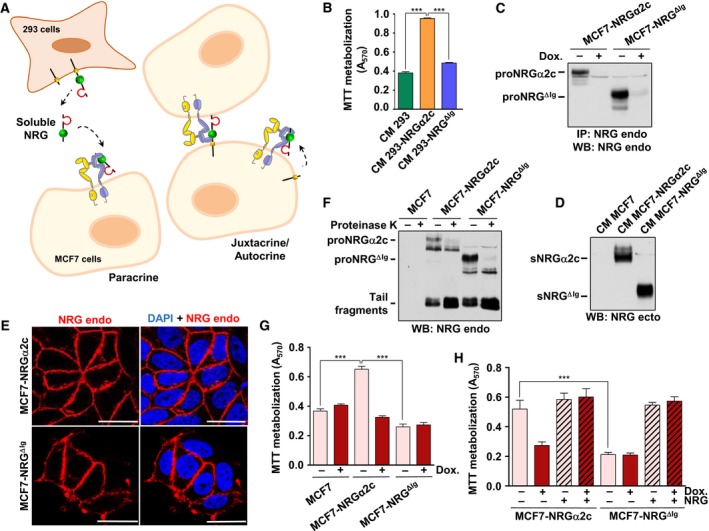
Biological activity of NRGα2c and NRG^ΔIg^. (A) Representation of the paracrine and juxtacrine/autocrine form of intercellular communication of NRG. In the paracrine form, the soluble NRG produced by 293 cells activates the HER receptors on MCF7 cells that reside at a certain distance from where the NRG is synthesized. In the juxtacrine form, the transmembrane NRG produced in MCF7 cells is able to active the HER receptors present at the membrane of cells that are in physical contact with the cell that produces the NRG. In the autocrine form, the soluble NRG produced in MCF7 cells is able to active HER receptors in the same cell that produced the NRG. (B) Effect of the conditioned medium of 293, 293‐NRGα2c, and 293‐NRG^ΔIg^ cells in proliferation. MCF7 cells were stimulated with the indicated conditioned medium, and MTT metabolization was measured 5 days later. ***P <* *0.001. (C) Expression of proNRGα2c and proNRG^ΔIg^ in MCF7tetoff cells. Clones of MCF7tetoff expressing proNRGα2c and proNRG^ΔIg^ (MCF7‐NRGα2c and MCF7‐NRG^ΔIg^) were cultured with or without doxycycline (DOX, 10 ng·mL^−1^) during 2 days and then lysed. The samples were analyzed by western blotting with the anti‐endodomain antibody. (D) The levels of sNRG released in the conditioned medium of MCF7‐NRGα2c and MCF7‐NRG^ΔIg^ cells were analyzed by western blotting with the anti‐ectodomain antibody. (E) Subcellular distribution of proNRGα2c and proNRG^ΔIg^ in MCF7 cells was analyzed by immunofluorescence using the anti‐endodomain antibody. Nuclear staining was performed with DAPI. Images were captured using a Leica TCS SP5 confocal microscope. Bar, 25 μm. (F) Protease protection experiments. Intact MCF7tetoff cells expressing proNRGα2c and proNRG^ΔIg^ were treated with or without proteinase K and lysed. The samples were analyzed by western blotting with the anti‐endodomain antibody. (G) Proliferation of MCF7, MCF7‐NRGα2c, and MCF7‐NRG^ΔIg^ cells. These cells were cultured in the presence or absence of doxycycline (10 ng·mL^−1^), and MTT metabolization was measured 5 days later. ***P <* *0.001. (H) Cells cultured in the presence or absence of doxycycline (10 ng·mL^−1^) were treated with or without NRG 10 nm, and the MTT metabolization was measured 5 days later. ***P <* *0.001.

The effect of NRGα2c or NRG^ΔIg^ on cell proliferation was investigated using different experimental settings aimed at exploring paracrine, juxtacrine and autocrine modes of intercellular communication (Fig. 4A). The paracrine model consisted in the addition to MCF7 cells of conditioned media from 293 cells expressing proNRGα2c or proNRG^ΔIg^. The negative control for these experiments was conditioned medium from 293 cells that do not express NRGs. As shown in Fig. 4B, conditioned media from 293 cells expressing proNRGα2c provoked an increase in the proliferation of MCF7 cells, measured using MTT metabolization assays. In contrast, MCF7 cells incubated with conditioned media from 293 cells expressing proNRG^ΔIg^ grew much less, with their MTT values slightly higher than those of MCF7 cells incubated with the media from parental 293 cells. These results indicate that the paracrine action of sNRGα2c in terms of stimulation of cell proliferation is superior to that of sNRG^ΔIg^.

The second model used consisted in MCF7 cells expressing proNRGα2c or proNRG^ΔIg^. Such model consists of a single cell type that produces the transmembrane growth factor as well as the whole machinery for the transduction of proliferative signals. It therefore allows the assessment of autocrine/paracrine as well as juxtacrine properties of proNRGs (Fig. 4A). For these experiments, MCF7^tetoff^ cells were transfected with the cDNA coding for wild‐type proNRGα2c or proNRG^ΔIg^, subcloned into the pRevTRE mammalian expression vector. This system allows the regulated expression of proteins under the control the tetracycline transactivator. Several clones of MCF7‐NRGα2c and MCF7‐NRG^ΔIg^ cells were isolated, and Fig. 4C shows the level of expression of proNRGα2c and proNRG^ΔIg^ and their repression by doxycycline in two clones selected for their analogous expression of both proNRGs. These clones released similar amounts of sNRGα2c or sNRG^ΔIg^ to the culture media (Fig. 4D). Immunofluorescence experiments showed that proNRGα2c and proNRG^ΔIg^ were located at the plasma membrane of MCF7 cells (Fig. 4E) and colocalized with HER2 and HER3 (data not shown). Likewise, when the MCF7‐NRGα2c and MCF7‐NRG^ΔIg^ cells were incubated with proteinase K, the slowest mobility band of each form, which represents the mature cell surface‐exposed proNRG, was proteolytically processed (Fig. 4F), and this was accompanied by an increase in the amount of the cell‐bound truncated tail fragments. These results are similar to those obtained in 293 cells (Fig. 1) and demonstrate that wild‐type proNRGα2c and proNRG^ΔIg^ reach the plasma membrane and are cleaved to generate soluble forms of the factor.

Expression of wild‐type proNRGα2c favored proliferation of MCF7‐NRGα2c cells, and such effect was sensitive to doxycycline (Fig. 4G). Proliferation of MCF7‐NRGα2c cells treated with doxycycline was similar to that of parental, untransfected MCF7 cells. MCF7‐NRG^ΔIg^ proliferated less than MCF7‐NRGα2c cells, and that proliferation was insensitive to addition of doxycycline. MCF7‐NRGα2c and MCF7‐NRG^ΔIg^ proliferated similarly in response to addition of exogenous NRG (Fig. 4H), demonstrating that the differences in cell proliferation found between the cell lines were not due to differences in their proliferative capabilities.

### 3.6 HER pathway activation in MCF7‐NRGα2c and MCF7‐NRG^ΔIg^ cells and sensitivity to trastuzumab

**Figure 5:**
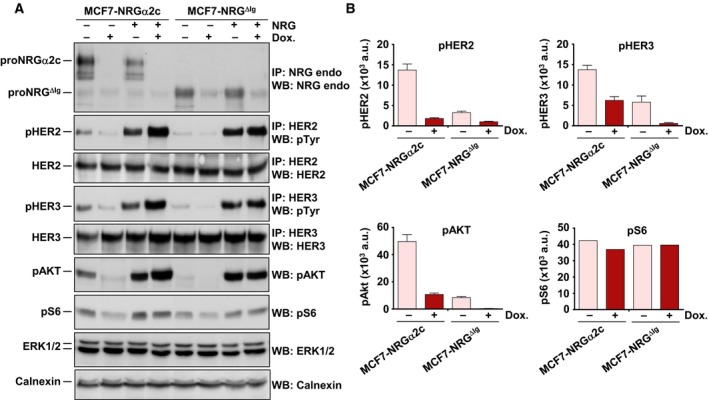
Cell surface NRGα2c activates signaling through ErbB receptors. (A) MCF7‐NRGα2c and MCF7‐NRG^ΔIg^ cells cultured in the presence or absence of doxycycline (10 ng·mL^−1^) and treated with or without NRG (10 nm) during 15 min were lysed. Immunoprecipitation (where pertinent) and western blotting were performed with the indicated antibodies. (B) Graphical representation of the quantification of the phosphorylation of HER2, HER3, AKT, and S6. Data are expressed as the mean ± SD of two independent experiments, except for the phosphorylation of S6, performed as in B.

The signaling capability of wild‐type proNRGα2c and proNRG^ΔIg^ in MCF7 cells expressing these forms was then analyzed. Resting levels of pHER2, pHER3, pAKT, and pS6 were higher in MCF7‐NRGα2c than in MCF7‐NRG^ΔIg^ cells (Fig. 5A,B). Repression of NRG expression by the addition of doxycycline reduced the phosphorylation status of all these proteins, especially in MCF7‐NRGα2c cells. Both cell lines responded to the addition of exogenous NRG, indicating that they preserved signaling responses to the added growth factor.

**Figure 6:**
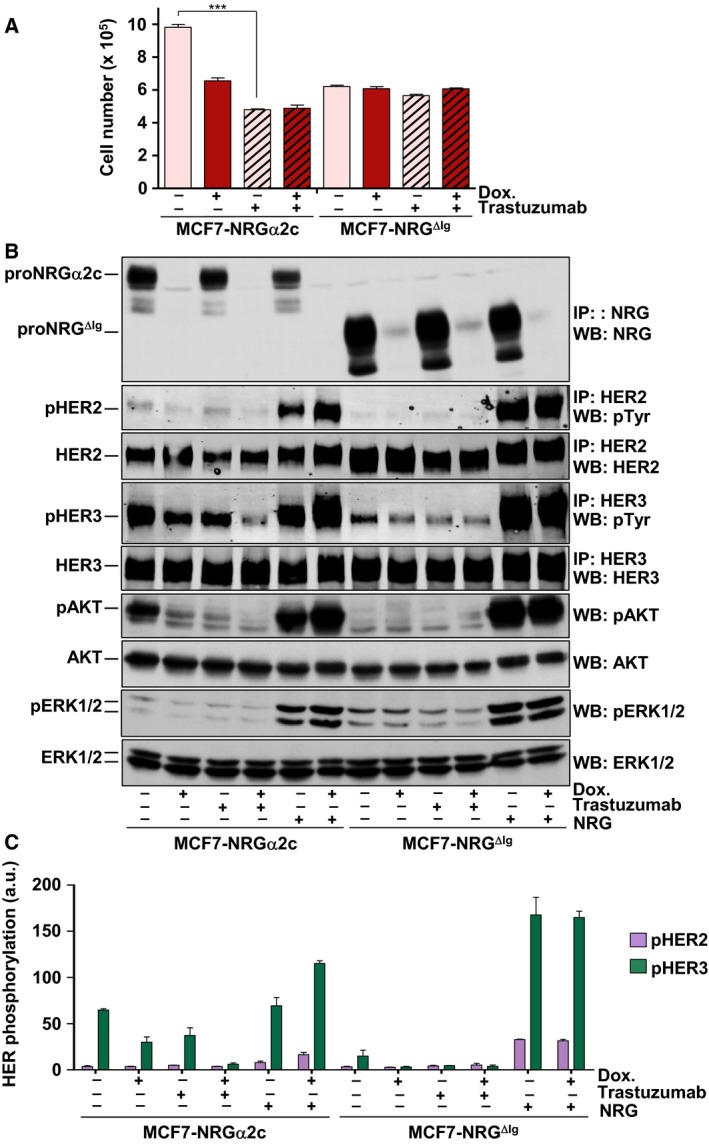
MCF7‐NRGα2c cells are sensitive to the anti‐HER2 therapeutic antibody trastuzumab. (A) Effect of trastuzumab in the proliferation of MCF7‐NRGα2c and MCF7‐NRG^ΔIg^. Cells cultured in the presence or absence of doxycycline (10 ng·mL^−1^) were treated with or without trastuzumab (50 nm), and the number of the cells was counted 5 days later. ***P <* *0.001. (B) MCF7‐NRGα2c and MCF7‐NRG^ΔIg^ cells cultured in the presence or absence of doxycycline (10 ng·mL^−1^) and treated with or without trastuzumab (50 nm) and NRG (10 nm) during 15 min. The cells were lysed and the expression of different proteins was analyzed by western blotting with the indicated antibodies. (C) Graphical representation of the quantification of the phosphorylation of HER2 and HER3. Data are expressed as the mean ± SD of three independent experiments performed as in B.

The sensitivity of MCF7‐NRGα2c and MCF7‐NRG^ΔIg^ cells to the anti‐HER2 therapeutic antibody trastuzumab was then explored. Trastuzumab has been formerly reported to exert a potent inhibition of proliferation of NRG‐expressing cells (Yuste *et al*., 2005). Moreover, clinical responses to trastuzumab have been shown to correlate with the expression of NRGs in breast cancer tumors which do not overexpress HER2 (de Alava *et al*., 2007). Trastuzumab substantially decreased proliferation of MCF7‐NRGα2c cells (Fig. 6A). Such decrease in cell proliferation was at least the magnitude obtained by repressing the expression of proNRGα2c by treatment with doxycycline. Combination of trastuzumab treatment with repression of the expression of proNRGα2c did not augment the inhibition of cell proliferation caused by trastuzumab alone. These data indicated that trastuzumab neutralized the proliferation advantage supplied by the expression of proNRGα2c in MCF7 cells.

Trastuzumab slightly decreased the proliferation of MCF7‐NRG^ΔIg^ cells. Addition of doxycycline or combination of the latter with trastuzumab did not substantially change the proliferation of these cells when compared to that of untreated MCF7‐NRG^ΔIg^ cells.

Western blotting (Fig. 6B) and quantitative analyses (Fig. 6C) demonstrated that trastuzumab inhibited the degree of constitutive activation of HER3, and AKT present in MCF‐NRGα2c cells. Interestingly, the levels of pHER2 were poorly affected by trastuzumab in MCF7‐NRGα2c cells. Trastuzumab also decreased pHER3 levels in MCF7‐NRG^ΔIg^ cells. However, an action of trastuzumab on the activation status of the other proteins analyzed (pHER2 and pAKT) could not be adequately interpreted as their degree of activation was very low in MCF7‐NRG^ΔIg^ cells (Fig. 6B,C). The levels of pERK1/2 were low in both cell lines and were not affected by trastuzumab or doxycycline. We also tested the action of pertuzumab, an antibody that inhibits ligand‐dependent HER2 receptor dimerization (Agus *et al*., 2002), on pHER2, pHER3, and downstream signaling. These experiments reported similar results to those obtained with trastuzumab (Fig. S1). However, it should be noted that pertuzumab decreased pHER2 levels in MCF7‐NRGα2c cells.

## 4 Discussion

The accumulation of reports that link NRGs to cancer justifies studies aimed at deciphering the relevance of different regions of NRGs in their biological activity, not only because of that, but also because such studies may uncover new therapeutic possibilities. In fact, some reports have already approached that latter possibility, showing the therapeutic benefit of acting on the NRG‐ErbB system (Jones *et al*., 2017). While some studies have offered information on the role of different domains in the sorting of NRGs (Montero *et al*., 2007, 2011), the impact of elimination of the different domains on their biological activity has not been explored and had to be done. With that purpose in mind, we prepared several deletion mutants of proNRGα2c, a prototypical proNRG, and analyzed their biological activity, measured as capability to activate HER receptors and proliferation responses.

We observed that the Ig‐like domain of proNRGs exerts an important role in their signal‐promotion capabilities, but did not affect cell surface sorting. In fact, elimination of the Ig‐like region did not affect transport and accumulation of the mutant proNRG^ΔIg^ at the cell surface, as indicated by immunofluorescence or proteinase K protection experiments. Moreover, regulated proteolytic cleavage, which exclusively occurs at the cell surface (Montero *et al*., 2007), was unaffected by deletion of the Ig‐like domain, not only indicating that such domain is dispensable for proteolytic cleavage, but also supporting the fact that proNRG^ΔIg^ reached the cell surface as well as wild‐type proNRGα2c. That such cleavage occurred similarly to that of the wild‐type protein was also suggested by the accumulation of sNRG^ΔIg^ in the culture media, in amounts similar to those observed in the media of proNRGα2c‐expressing cells.

Initially surprising was the fact that conditioned media from 293 cells expressing proNRG^ΔIg^ were much less efficient in promoting tyrosine phosphorylation of HER receptors. Such effect was accompanied by decreased phosphorylation of several proteins that are used as readouts of activation of pathways that participate in NRG–HER signal transduction. As a final consequence, the much lower activation of signaling by this system translated into poor stimulation of cell proliferation by NRG^ΔIg^ with respect to wild‐type NRGα2c.

The reason for the failure of soluble or transmembrane NRG^ΔIg^ to efficiently activate HER2 and HER3 is unknown. Former reports indicated that the Ig‐like domain is required for efficient interaction of the EGF‐like domain of NRGs with their receptors (Eto *et al*., 2006). Such phenomenon can be due to a direct action of the Ig‐like region of NRGs on receptor binding or a more global change in the structure affecting the EGF‐like region, and ultimately altering interaction with HER receptors. While this appears possible, it is worth mentioning that other growth factors that belong to the EGF family of transmembrane growth factors, such as proTGFα, are devoid of Ig‐like domains and their soluble form is biologically active (Massague and Pandiella, 1993). It is also interesting to mention that most commercially available forms of NRGs are prepared without the Ig‐like domain and are active. It is possible that the amounts used are much higher than the amounts we used from the culture media and that may explain the weak stimulation properties of the media form proNRG^ΔIg^‐transfected cells as compared to the wild‐type or even to the commercially available NRG.

Using the MCF7 reporter system, we observed that activation of HER3, measured as its tyrosine phosphorylation, was more efficiently induced by sNRGα2c with respect to sNRG^ΔIg^. Such differential action may be due to several circumstances. Tyrosine phosphorylation of HER receptors has been shown to occur in an asymmetric manner, in which a donor receptor phosphorylates tyrosine residues of an adjacent acceptor receptor (Zhang *et al*., 2006). In the case of HER2–HER3 receptor dimers, and due to the deficient kinase activity of HER3 (Shi *et al*., 2010), the latter is expected to mainly act as the acceptor receptor, being HER2 the donor kinase. The restricted kinase activity of HER3 may very well explain the poor tyrosine phosphorylation of HER2, as indicated by western blotting experiments with anti‐HER2 phosphospecific antibodies. Moreover, previously published results (Sanchez‐Martin and Pandiella, 2012) have already shown that the pY signal obtained in western blots in which HER2 was immunoprecipitated after stimulation with NRG could be due to pY‐HER3 which coprecipitated with HER2.

As proliferation of MCF7 cells has been reported to be insensitive to the action of trastuzumab (Agus *et al*., 2002; Yuste *et al*., 2005), it is interesting that this drug had an effect on the proliferation of MCF7‐NRGα2c cells. While such finding has already been reported (Yuste *et al*., 2005), the responsible mechanism of action was not explored. Interestingly, the amount of pHER2 present in MCF7‐NRGα2c cells was poorly affected by trastuzumab, a finding that falls in line with published data using soluble NRG to activate HER2 (Agus *et al*., 2002). In contrast, trastuzumab has a substantial effect on the tyrosine phosphorylation of HER3, and on the activation status of AKT. Those findings are relevant, given the important role of the HER3–PI3K signaling route in the proliferation of breast cancer cells (Chakrabarty *et al*., 2012). Our results, together with data available in the literature, led us to postulate that the antiproliferative action of trastuzumab on MCF7‐NRGα2c cells may occur indirectly, by decreasing the HER2‐mediated tyrosine phosphorylation of HER3, which in turn affects HER3‐dependent downstream signaling.

An important aspect that requires some comments is to which extent the results herewith reported may have a translational impact. The finding that the Ig‐like domain of proNRGs favors their HER receptor activating properties opens the possibility of targeting that domain to improve the effectiveness of anti‐NRG therapies. Due to the unquestionable capability of NRGs to promote proliferation of cells bearing HER receptors, neutralization of NRG activity may represent an attractive approach (Jones *et al*., 2017; Montero *et al*., 2007, 2011; Yuste *et al*., 2005). One could envisage several scenarios in which neutralization of NRGs may be of potential therapeutic benefit. On the one hand, neutralizing anti‐NRG antibodies may reduce proliferation of tumors in which NRGs fed tumoral cells expressing HER receptors. Moreover, the expression of NRGs by some tumoral tissues may offer additional novel therapeutic possibilities, some of them based on their structural and cell biological properties. Thus, the fact that NRGs are synthesized as transmembrane molecules offers the possibility of raising antibodies that recognize the extracellular region of the proNRGs. These antibodies may not only neutralize the growth‐promoting properties of NRGs, but may also serve to generate immune responses against the tumor, similarly to those raised against therapies that target the HER receptors (Clynes *et al*., 2000). Furthermore, considering the transmembrane nature of proNRGs, they could preserve properties similar to those of other transmembrane proteins, such as HER receptors, including internalization and recycling (Sorkin and Goh, 2009). Given the fact that some efficient treatments directed to those receptors include antibody–drug conjugates that internalize and deliver a cytotoxic drug, the possibility of using a similar approach to target tumoral cells expressing proNRGs appears attractive and should be explored.

## 5 Conclusion

In conclusion, this report demonstrates that the Ig‐like region of proNRGs exerts an important role in their capability to activate mitogenic responses upon ErbB/HER receptor activation. This knowledge is important when considering the potential targeting of proNRGs in tumors, as it may improve the antiproliferative properties of agents aimed at neutralizing the pro‐oncogenic properties of proNRGs.

## Author contributions

AC and JCM performed experiments, prepared figures, and wrote parts of the manuscript. RR‐B performed experiments. AP supervised research and wrote parts of the manuscript. All authors corrected and approved the final version of the manuscript.

## Supporting information


**Fig. S1.** MCF7‐NRGα2c cells are sensitive to the anti‐HER2 therapeutic antibody pertuzumab.

## References

[mol212310-bib-0001] Agus DB , Akita RW , Fox WD , Lewis GD , Higgins B , Pisacane PI , Lofgren JA , Tindell C , Evans DP , Maiese K *et al* (2002) Targeting ligand‐activated ErbB2 signaling inhibits breast and prostate tumor growth. Cancer Cell 2, 127–137. 12204533 10.1016/s1535-6108(02)00097-1

[mol212310-bib-0002] de Alava E , Ocana A , Abad M , Montero JC , Esparis‐Ogando A , Rodriguez CA , Otero AP , Hernandez T , Cruz JJ and Pandiella A (2007) Neuregulin expression modulates clinical response to trastuzumab in patients with metastatic breast cancer. J Clin Oncol 25, 2656–2663. 17602072 10.1200/JCO.2006.08.6850

[mol212310-bib-0003] Atlas E , Cardillo M , Mehmi I , Zahedkargaran H , Tang C and Lupu R (2003) Heregulin is sufficient for the promotion of tumorigenicity and metastasis of breast cancer cells in vivo. Mol Cancer Res 1, 165–175. 12556556

[mol212310-bib-0004] Breuleux M (2007) Role of heregulin in human cancer. Cell Mol Life Sci 64, 2358–2377. 17530167 10.1007/s00018-007-7120-0 PMC11138466

[mol212310-bib-0005] Britsch S , Li L , Kirchhoff S , Theuring F , Brinkmann V , Birchmeier C and Riethmacher D (1998) The ErbB2 and ErbB3 receptors and their ligand, neuregulin‐1, are essential for development of the sympathetic nervous system. Genes Dev 12, 1825–1836. 9637684 10.1101/gad.12.12.1825 PMC316903

[mol212310-bib-0006] Burden S and Yarden Y (1997) Neuregulins and their receptors: a versatile signaling module in organogenesis and oncogenesis. Neuron 18, 847–855. 9208852 10.1016/s0896-6273(00)80324-4

[mol212310-bib-0007] Carraway KL III and Burden SJ (1995) Neuregulins and their receptors. Curr Opin Neurobiol 5, 606–612. 8580712 10.1016/0959-4388(95)80065-4

[mol212310-bib-0008] Chakrabarty A , Sanchez V , Kuba MG , Rinehart C and Arteaga CL (2012) Feedback upregulation of HER3 (ErbB3) expression and activity attenuates antitumor effect of PI3K inhibitors. Proc Natl Acad Sci USA 109, 2718–2723. 21368164 10.1073/pnas.1018001108 PMC3286932

[mol212310-bib-0009] Clynes RA , Towers TL , Presta LG and Ravetch JV (2000) Inhibitory Fc receptors modulate in vivo cytotoxicity against tumor targets. Nat Med 6, 443–446. 10742152 10.1038/74704

[mol212310-bib-0010] Ebbing EA , Medema JP , Damhofer H , Meijer SL , Krishnadath KK , van Berge Henegouwen MI , Bijlsma MF and van Laarhoven HW (2016) ADAM10‐mediated release of heregulin confers resistance to trastuzumab by activating HER3. Oncotarget 7, 10243–10254. 26863569 10.18632/oncotarget.7200 PMC4891117

[mol212310-bib-0011] Esparis‐Ogando A , Diaz‐Rodriguez E , Montero JC , Yuste L , Crespo P and Pandiella A (2002) Erk5 participates in neuregulin signal transduction and is constitutively active in breast cancer cells overexpressing ErbB2. Mol Cell Biol 22, 270–285. 11739740 10.1128/MCB.22.1.270-285.2002 PMC134212

[mol212310-bib-0012] Esparis‐Ogando A , Montero JC , Arribas J , Ocana A and Pandiella A (2016) Targeting the EGF/HER ligand‐receptor system in cancer. Curr Pharm Des 22, 5887–5898. 27426127 10.2174/1381612822666160715132233

[mol212310-bib-0013] Eto K , Eda K , Kanemoto S and Abe S (2006) The immunoglobulin‐like domain is involved in interaction of neuregulin1 with ErbB. Biochem Biophys Res Commun 350, 263–271. 17007820 10.1016/j.bbrc.2006.09.028

[mol212310-bib-0014] Falls DL (2003) Neuregulins: functions, forms, and signaling strategies. Exp Cell Res 284, 14–30. 12648463 10.1016/s0014-4827(02)00102-7

[mol212310-bib-0015] Hayes NV , Blackburn E , Smart LV , Boyle MM , Russell GA , Frost TM , Morgan BJ , Baines AJ and Gullick WJ (2007) Identification and characterization of novel spliced variants of neuregulin 4 in prostate cancer. Clin Cancer Res 13, 3147–3155. 17545517 10.1158/1078-0432.CCR-06-2237

[mol212310-bib-0016] Holmes WE , Sliwkowski MX , Akita RW , Henzel WJ , Lee J , Park JW , Yansura D , Abadi N , Raab H , Lewis GD *et al* (1992) Identification of heregulin, a specific activator of p185erbB2. Science 256, 1205–1210. 1350381 10.1126/science.256.5060.1205

[mol212310-bib-0017] Jeong H , Kim J , Lee Y , Seo JH , Hong SR and Kim A (2014) Neuregulin‐1 induces cancer stem cell characteristics in breast cancer cell lines. Oncol Rep 32, 1218–1224. 25018110 10.3892/or.2014.3330

[mol212310-bib-0018] Jones MR , Lim H , Shen Y , Pleasance E , Ch'ng C , Reisle C , Leelakumari S , Zhao C , Yip S , Ho J *et al* (2017) Successful targeting of the NRG1 pathway indicates novel treatment strategy for metastatic cancer. Ann Oncol 28, 3092–3097. 28950338 10.1093/annonc/mdx523

[mol212310-bib-0019] Kim J , Jang SJ , Choi CM and Ro JY (2016) Correlation of histologic subtypes and molecular alterations in pulmonary adenocarcinoma: therapeutic and prognostic implications. Adv Anat Pathol 23, 330–338. 27403614 10.1097/PAP.0000000000000121

[mol212310-bib-0020] Krane IM and Leder P (1996) NDF/heregulin induces persistence of terminal end buds and adenocarcinomas in the mammary glands of transgenic mice. Oncogene 12, 1781–1788. 8622899

[mol212310-bib-0021] Loeb JA and Fischbach GD (1995) ARIA can be released from extracellular matrix through cleavage of a heparin‐binding domain. J Cell Biol 130, 127–135. 7540614 10.1083/jcb.130.1.127 PMC2120519

[mol212310-bib-0022] Loeb JA , Khurana TS , Robbins JT , Yee AG and Fischbach GD (1999) Expression patterns of transmembrane and released forms of neuregulin during spinal cord and neuromuscular synapse development. Development 126, 781–791. 9895325 10.1242/dev.126.4.781

[mol212310-bib-0023] Massague J and Pandiella A (1993) Membrane‐anchored growth factors. Annu Rev Biochem 62, 515–541. 8394682 10.1146/annurev.bi.62.070193.002503

[mol212310-bib-0024] Meetze K , Vincent S , Tyler S , Mazsa EK , Delpero AR , Bottega S , McIntosh D , Nicoletti R , Winston WM , Weiler S *et al* (2015) Neuregulin 1 expression is a predictive biomarker for response to AV‐203, an ERBB3 inhibitory antibody, in human tumor models. Clin Cancer Res 21, 1106–1114. 25542901 10.1158/1078-0432.CCR-14-2407

[mol212310-bib-0025] Mei L and Xiong WC (2008) Neuregulin 1 in neural development, synaptic plasticity and schizophrenia. Nat Rev Neurosci 9, 437–452. 18478032 10.1038/nrn2392 PMC2682371

[mol212310-bib-0026] Meyer D and Birchmeier C (1995) Multiple essential functions of neuregulin in development. Nature 378, 386–390. 7477375 10.1038/378386a0

[mol212310-bib-0027] Montero JC , Rodriguez‐Barrueco R , Ocana A , Diaz‐Rodriguez E , Esparis‐Ogando A and Pandiella A (2008) Neuregulins and cancer. Clin Cancer Res 14, 3237–3241. 18519747 10.1158/1078-0432.CCR-07-5133

[mol212310-bib-0028] Montero JC , Rodriguez‐Barrueco R and Pandiella A (2011) Transautocrine signaling by membrane neuregulins requires cell surface targeting, which is controlled by multiple domains. J Biol Chem 286, 24350–24363. 21572038 10.1074/jbc.M110.190835 PMC3129214

[mol212310-bib-0029] Montero JC , Rodriguez‐Barrueco R , Yuste L , Juanes PP , Borges J , Esparis‐Ogando A and Pandiella A (2007) The extracellular linker of pro‐neuregulin‐alpha2c is required for efficient sorting and juxtacrine function. Mol Biol Cell 18, 380–393. 17108327 10.1091/mbc.E06-06-0511 PMC1783780

[mol212310-bib-0030] Montero JC , Yuste L , Diaz‐Rodriguez E , Esparis‐Ogando A and Pandiella A (2000) Differential shedding of transmembrane neuregulin isoforms by the tumor necrosis factor‐alpha‐converting enzyme. Mol Cell Neurosci 16, 631–648. 11083924 10.1006/mcne.2000.0896

[mol212310-bib-0031] Montero JC , Yuste L , Diaz‐Rodriguez E , Esparis‐Ogando A and Pandiella A (2002) Mitogen‐activated protein kinase‐dependent and ‐independent routes control shedding of transmembrane growth factors through multiple secretases. Biochem J 363, 211–221. 11931648 10.1042/0264-6021:3630211 PMC1222469

[mol212310-bib-0032] Sanchez‐Martin M and Pandiella A (2012) Differential action of small molecule HER kinase inhibitors on receptor heterodimerization: therapeutic implications. Int J Cancer 131, 244–252. 21826647 10.1002/ijc.26358

[mol212310-bib-0033] Schwarz LJ , Hutchinson KE , Rexer BN , Estrada MV , Gonzalez Ericsson PI , Sanders ME , Dugger TC , Formisano L , Guerrero‐Zotano A , Red‐Brewer M *et al* (2017) An ERBB1‐3 neutralizing antibody mixture with high activity against drug‐resistant HER2+ breast cancers with ERBB ligand overexpression. J Natl Cancer Inst 109, https://doi.org/10.1093/jnci/djx065. 10.1093/jnci/djx065 PMC6543617 29059433

[mol212310-bib-0034] Seoane S , Montero JC , Ocana A and Pandiella A (2016) Breast cancer dissemination promoted by a neuregulin‐collagenase 3 signalling node. Oncogene 35, 2756–2765. 26364598 10.1038/onc.2015.337

[mol212310-bib-0035] Shi F , Telesco SE , Liu Y , Radhakrishnan R and Lemmon MA (2010) ErbB3/HER3 intracellular domain is competent to bind ATP and catalyze autophosphorylation. Proc Natl Acad Sci USA 107, 7692–7697. 20351256 10.1073/pnas.1002753107 PMC2867849

[mol212310-bib-0036] Sorkin A and Goh LK (2009) Endocytosis and intracellular trafficking of ErbBs. Exp Cell Res 315, 683–696. 19278030 10.1016/j.yexcr.2008.07.029 PMC2605728

[mol212310-bib-0037] Tsai MS , Shamon‐Taylor LA , Mehmi I , Tang CK and Lupu R (2003) Blockage of heregulin expression inhibits tumorigenicity and metastasis of breast cancer. Oncogene 22, 761–768. 12569369 10.1038/sj.onc.1206130

[mol212310-bib-0038] Warren CM , Kani K and Landgraf R (2006) The N‐terminal domains of neuregulin 1 confer signal attenuation. J Biol Chem 281, 27306–27316. 16825199 10.1074/jbc.M512887200

[mol212310-bib-0039] Wen D , Suggs SV , Karunagaran D , Liu N , Cupples RL , Luo Y , Janssen AM , Ben‐Baruch N , Trollinger DB , Jacobsen VL *et al* (1994) Structural and functional aspects of the multiplicity of Neu differentiation factors. Mol Cell Biol 14, 1909–1919. 7509448 10.1128/mcb.14.3.1909-1919.1994 PMC358549

[mol212310-bib-0040] Yang L , Li Y , Shen E , Cao F , Li L , Li X , Wang X , Kariminia S , Chang B , Li H *et al* (2017) NRG1‐dependent activation of HER3 induces primary resistance to trastuzumab in HER2‐overexpressing breast cancer cells. Int J Oncol 51, 1553–1562. 29048656 10.3892/ijo.2017.4130

[mol212310-bib-0041] Yuste L , Montero JC , Esparis‐Ogando A and Pandiella A (2005) Activation of ErbB2 by overexpression or by transmembrane neuregulin results in differential signaling and sensitivity to herceptin. Cancer Res 65, 6801–6810. 16061662 10.1158/0008-5472.CAN-04-4023

[mol212310-bib-0042] Zhang X , Gureasko J , Shen K , Cole PA and Kuriyan J (2006) An allosteric mechanism for activation of the kinase domain of epidermal growth factor receptor. Cell 125, 1137–1149. 16777603 10.1016/j.cell.2006.05.013

