# Supplementary material for: The immunoglobulin‐like domain of neuregulins potentiates ErbB3/HER3 activation and cellular proliferation
Source: Mol Oncol. 2018 May 14;12(7):1061–76. doi: 10.1002/1878-0261.12310 (PMC6026874; doi:10.1002/1878-0261.12310)
Supplement: Supplementary file 1 — Fig. S1. MCF7‐NRGα2c cells are sensitive to the anti‐HER2 therapeutic antibody pertuzumab. [file MOL2-12-1061-s001.pdf]

Supplementary Figure S1

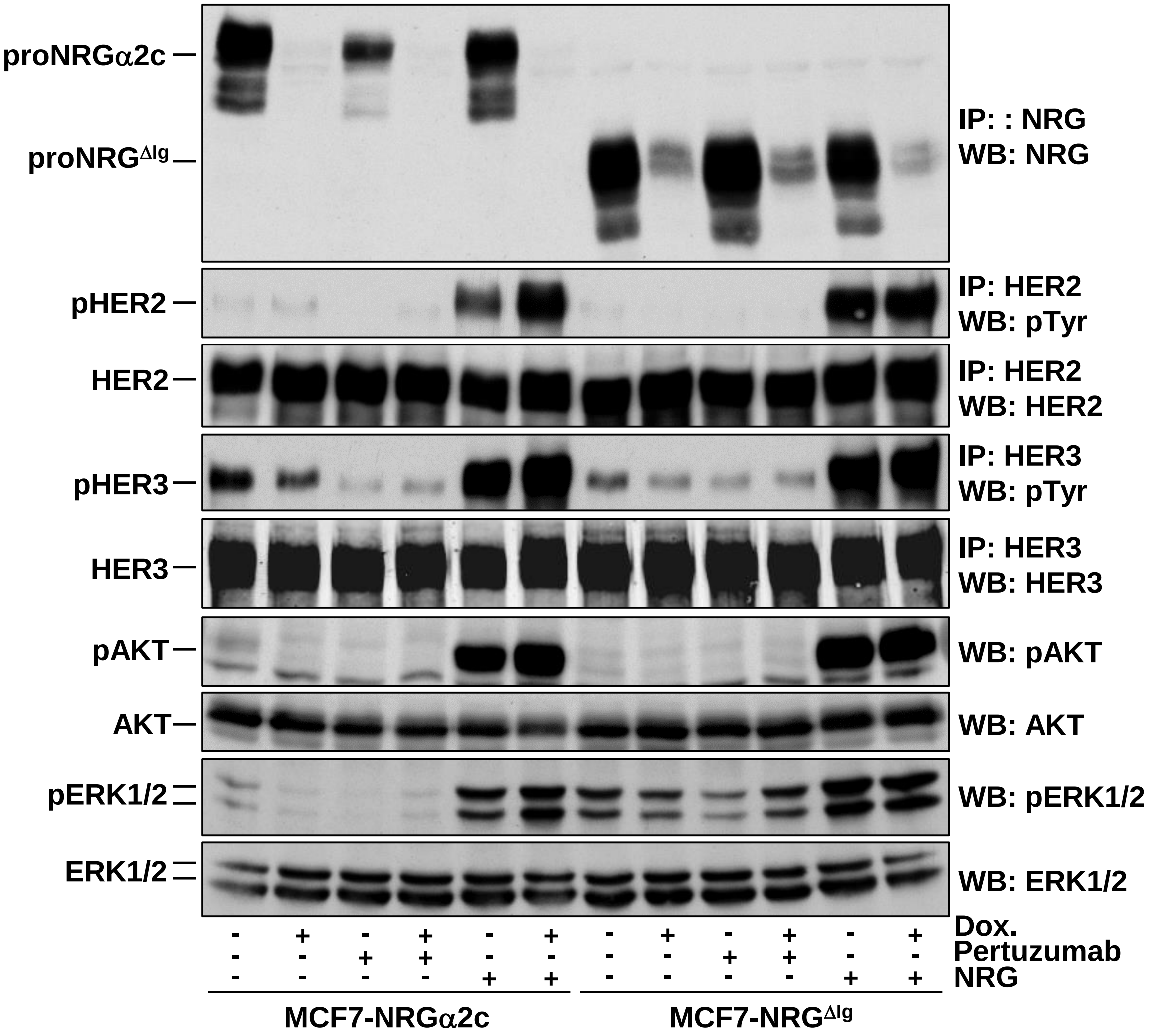

MCF7-NRGα2c cells are sensitive to the anti-HER2 therapeutic antibody pertuzumab.

MCF7-NRGα2c and MCF7-NRG$^{\Delta Ig}$ cells cultured in the presence or absence of doxycycline (10 ng/ml) and treated with or without pertuzumab (50 nM) and NRG (10 nM) during 15 minutes. The cells were lysed and the expression of different proteins was analysed by Western blot with the indicated antibodies.
